# Sliding twin-domains in self-heated needle-like VO_2_ single crystals

**DOI:** 10.1038/s41598-020-63694-1

**Published:** 2020-04-22

**Authors:** Bertina Fisher, Larisa Patlagan, George M. Reisner

**Affiliations:** Physics Department, Technion, Haifa, 32000 Israel

**Keywords:** Physics, Phase transitions and critical phenomena

## Abstract

The prototypical metal-insulator transition in VO_2_ at 340 K is from a high-temperature rutile phase to a low-temperature monoclinic phase. The lower symmetry of the monoclinic structure removes the degeneracy of the two equivalent directions of the tetragonal structure, giving rise to twin domains. Since formation of domain walls require energy most needle-like monoclinic single crystal are single-domain. The mixed metal-insulator state in self-heated needle-like single crystals exhibits various domain patterns, the most remarkable being static insulating triangular domains embedded in the metal and narrow insulating domains sliding along the metallic background in the direction of the electric current. Reported here are results obtained for some rare needle-like twinned VO_2_ single crystals. Such sample revealed a unique feature: joint static triangular twins emit sliding twin domains, first overlapping and later disjoining. Dark and bright twins and dim metallic background were seen for optimal orientation under a microscope, due to polarization by reflection.

## Introduction

Twin domains are expected to appear when a displacive transition occurs from a higher to a lower symmetry structure as is the case when VO_2_ is cooled through the transition at 340 K from the rutile (metallic) to the monoclinic (insulating) phase. Domain patterns of twins in the lower symmetry phase are visible under the optical microscope in reflected polarized light from surfaces of VO_2_ in the low- temperature, monoclinic phase^1^. This twinned structure showed up under crossed polarizers as bright (maximal reflectivity) and dark domains (reflected light is extinct) of orientations identified by X-ray diffraction. The same result may be obtained via polarization by reflection; this requires finding the optimal orientation of the sample with respect to the incident light, for maximal contrast between the two types of domains.

The mixed insulator-metal (I-M) phase can be induced by various routes such as heating by an external source, self-heating (e.g. Joule-heating) upon passage of an electric current through the sample, or laser irradiation and it depends on the morphology of the sample (single crystal, poly-crystal, thin film). I-M domain patterns in the mixed state of VO_2_ crystals are visible under the microscope due to the much higher reflectivity of the insulating phase relative to that of the metal. The most remarkable domain patterns observed in the mixed-state of VO_2_ single crystals are static insulating triangular domains^2–6^ embedded in the metallic background and narrow insulating domains sliding along the metallic background in the direction of the electric current^2,7^. The contrast between metallic and insulating domains viewed under the microscope is enhanced under optimal orientation leading to polarization by reflection^8,9^; the domain patterns so obtained show static or sliding, dark and bright insulating twins along a dim metallic background.

The mixed-phase, induced in VO_2_ single crystals by dc electric currents applied at ambient temperature, appears in the current controlled negative differential resistivity (CC-NDR) regime of the nonlinear I-V characteristic governed by self- heating. In this regime the I-V characteristic is stable for R_L_ ≥ | dV/dI|_max,_ where R_L_ is the load resistance^10^, and there is no abrupt switching^11^. In high quality needle-like VO_2_ single crystals (cross-section area < ~2 × 10^−5^ cm^2^) the steady state NDR regime is remarkable: while crossing from the insulating to the mixed phase I(V) is almost-, or perfectly- smooth, the dissipation power P(=IV) versus current bends and this bend is accompanied by the appearance of insulating domains sliding along metallic background in the direction of the current^11^. The narrow insulating sliding domains, inclined at 80° (and 100°) to the sample’s length, are emitted from an unstable I-M boundary inclined at 50° to the sample’s length (see e.g. Figure 2 in ref. ^2^). This sliding, known for a long time^2^, is governed by the exchange of Peltier heat and latent heat at the metal-insulator (M-I) and the insulator-metal (I-M) boundaries of the sliding domains. The sliding velocity u increases linearly with the current density J, where du/dJ = Π/L where Π is the Peltier coefficient of the couple at the boundary and L, the latent heat per unit volume. The highest value of Π measured on high quality VO_2_ single crystal^2^ is 0.34 V and L = 240 J/ cm^3^ (calculated from the latent heat per mole given in ref. ^12^) yielding Π/L = 0.00154 cm^3^/As. This behavior was observed in many samples originating from different growth runs, all un-twinned. The energetics of sliding domains’ emission is provided by the relation between the dissipated power (P) and the domains’ emission frequency (f); the energy per emitted domain is given by ΔE = dP/df.

Since twin-boundaries require energy, most needle-like monoclinic single crystal are single-domain. Unexpectedly, one of our batches which contained twinned needle-like VO_2_ crystals provided the opportunity to investigate the mixed M-I state in such samples. Here we report on the investigation of two samples from this batch. The temperature dependence of the resistance, R(T), was measured between room temperature and 370 K, before or after I(V) measurements; the samples were viewed under the microscope and their mixed M-I domain structure was recorded on videos at various stages during I-V tracing. The emission frequency of sliding domains (f) and, when attainable, their velocity (u) was measured from the videos.

## Results and Discussions

D6(6) was a long needle-like VO_2_ single crystal with a shiny surface and no visible defects along its length. It was designated to improving the contrast between the sliding insulating domains and the metallic background in the mixed M-I state obtained via self-heating. Twinning was detected in this crystal prior to applying current when following a few orientation trials, dark and bright domains appeared along its length. It was clear that the strong contrast between the reflected natural light from the two types of domains is due to polarization by reflection^8,9^. At an early stage of the I-V measurements the sample broke into two pieces of almost equal lengths. This provided the opportunity to distinguish between the effects of external heating (heat treatment) and self-heating (during I-V measurements) in almost identical samples. The two pieces were labeled D6(6a) and D6(6b); the ratio of their respective lengths was l(6a)/l(6b) = 0.77 (see Table 1); for the sake of comparison the investigation of D6(6a) started with I-V measurements while that of D6(6b) with slow R(T) measurements between 295 K and 370 K.

**Table 1:** Sample dimensions (length l, width w and thickness h), transition temperatures T_IMT_ and T_MIT_, activation energy Δ before IMT and after MIT, resistivity ρ at 300 K after MIT, du/dJ and notable observations.

Sample	l×w×h (cm^3^)	T_IMT_	T_MIT_	Δ(eV)		ρ(300 K) (Ωcm)	du/dJ (cm^3^ /As)	Notable observations
				before IMT	after MIT			
D6(6a)	0.10 × 0.006 × 0.0033						0.0004	Annihilation of sliding domains close to a static domain. Figure 3.
D6(6b)	0.13 × 0.006 × 0.0033	342	339	0.35	0.45	221	0.0002	Twin boundaries removed by slow heat treatment. Figure 1(c).
D6(7)	0.065× 0.0045× 0.0038	342	339	0.37	0.46	226	0.0015	Sliding twins emitted by static triangular twins. Figure 4(b).

**Figure 1:**
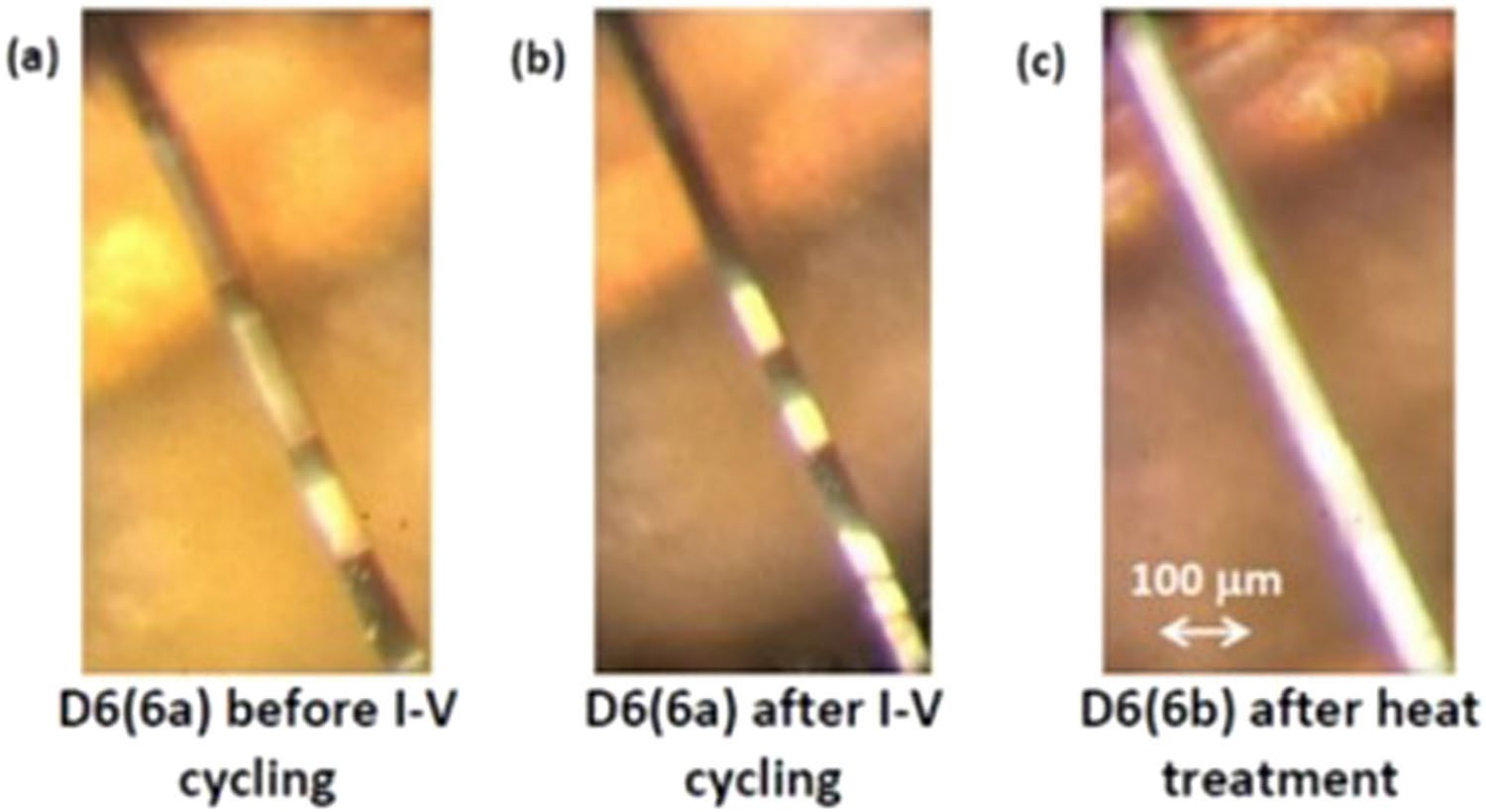
The effects of I-V cycling and of heat treatment on two pieces of sample D6(6). (**a**) The initially twinned domain structure of D6(6a); (**b**)the twined domain structure after I-V cycling; (**c**) the un- twinned domain structure of D6(6b) after heat treatment.

The sharp contrast between different twin domains is seen in the images of D6(6a) before and after I-V cycling (Fig. 1(a,b)). It is seen that the twin boundaries in this sample are somewhat shifted by I-V cycling but are not removed. The image of D6(6b) in Fig. 1(c) shows that the twin boundaries have been removed from this sample by slow heating up to 370 K and slow cooling while R(T) was measured (see below).

**Figure 2:**
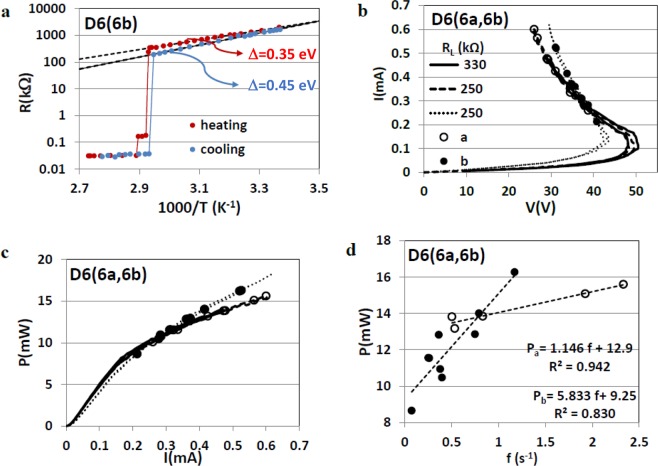
The effect of heat treatment of sample D6(6b) on its I-V characteristic and domain emission compared with those of the twinned sample D6(6a). (**a**) Resistance (R) versus inverse temperature for sample D6(6b), (**b**) I-V characteristics, (**c**)Power (P) versus current (I) and (**d**) P versus emission frequency (**f**) for both samples. In frames (**b,c)** solid and dashed lines correspond to sample D6(6a) and dotted line to D6(6b).

The traces R (1/1000 T) for heating and cooling of sample D6(6b) in the insulating state seen in Fig. 2(a) are different; the resistance is much larger and the activation energy Δ is much smaller upon heating than upon cooling. Also, the I-M transition is in two steps while the M-I transition is in one steep jump. The insulator around twin boundaries transits at higher temperature than the bulk^13^.

**Figure 3:**
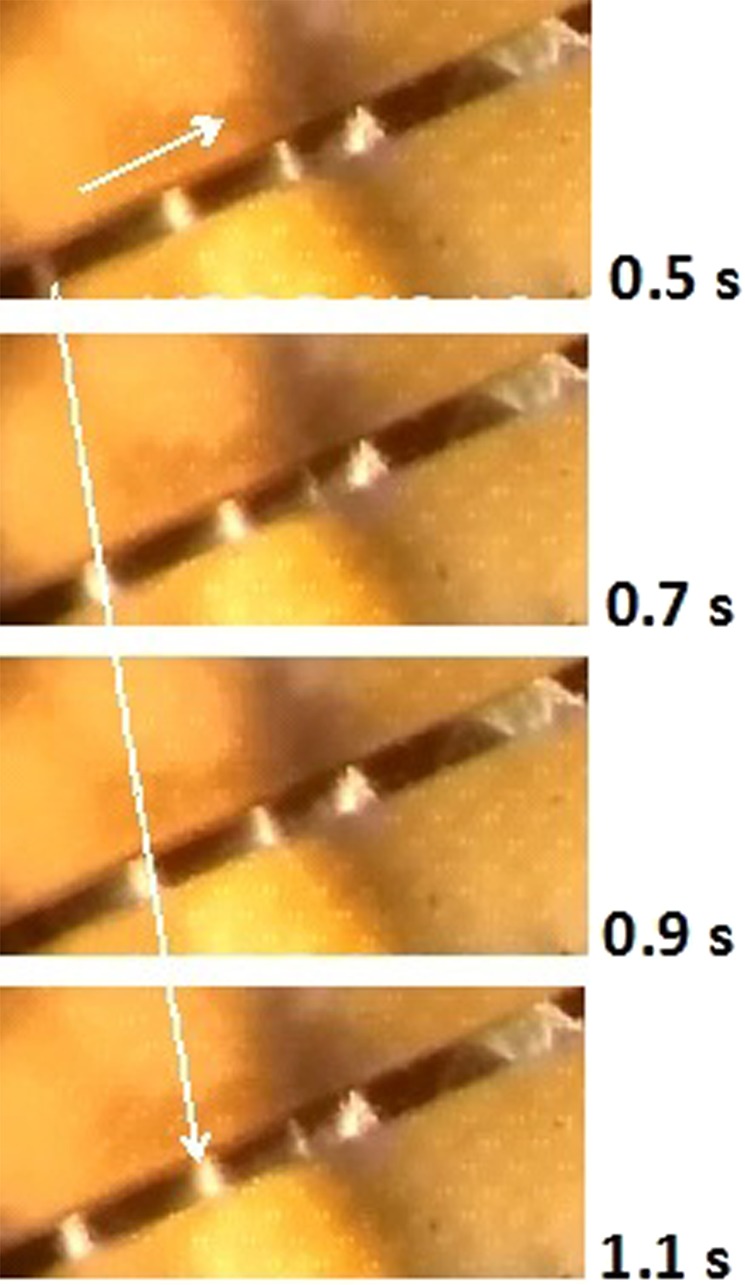
Annihilation of sliding domains in sample D6(6a) as they approach a static triangular domain. A sequence of images sniped from a video at instances 0.2 s apart shows domains sliding from left to right (see arrow) toward a static triangular domain; the domain closest to the triangle at t = 0.5 s has disappeared at t = 0.7 s; the one closest to the triangle at t = 0.9 s is being annihilated at t = 1.1 s (see video for I = 0.6 mA).

The d. c. I(V) traces for D6(6a) and D6(6b) shown in Fig. 2(b) are typical for current induced nonlinear conductivity in VO_2_ needles at ambient temperature, under steady state conditions. With increasing current, the voltage reached a maximum, V_max_, that marks the onset of NDR, which is significantly larger in the shorter D6(6a) sample (with twin boundaries) than in the longer D6(6b) sample (without twin boundaries). The transition to the mixed M- I state is smooth and is marked by bending of P(I) [Fig. 2(c)] and by the appearance of sliding domains; the open and filled circles on the V(I) and P(I) traces mark the points where dynamic phenomena were recorded on videos. Out of several tens of video clips recorded at different points on the I-V characteristics only a few showed, at fixed current, constant emission rates and even fewer showed constant velocities over short durations. The twinning in one sample and un-twinning in the second had very little effect on their mixed states. In both, the mixed state was irregular, consisting of a mixture of sliding and static domains; in the course of sliding the domains widened or shrank, split or coalesced with neighboring domains, or just disappeared. The dissipated power P(=IV) is plotted in Fig. 2(d) as function of emission frequency (f) for both D6(6a) and D6(6b). The fitted linear trend-lines show that the energy cost for domain emission in the twinned sample (D6(6a)) is lower than in the un-twinned one (D6(6b)): dP_a_/df < dP_b_/df. Rare data of u(J) obtained for both samples showed du/dJ values much lower than those obtained in the past in single domain samples. The irregular mixed state in both samples and low du/dJ are probably due to structural defects in the samples. The tendency of twinning may have also been promoted by such defects. Figure 3 is an example of the irregular mixed state. Four images snipped from a video clip recorded during I(V) measurements on D6(6a) at instances 0.2 seconds apart show domains sliding from left to right and disappearing successively as they approach a static triangular domain.

**Figure 4:**
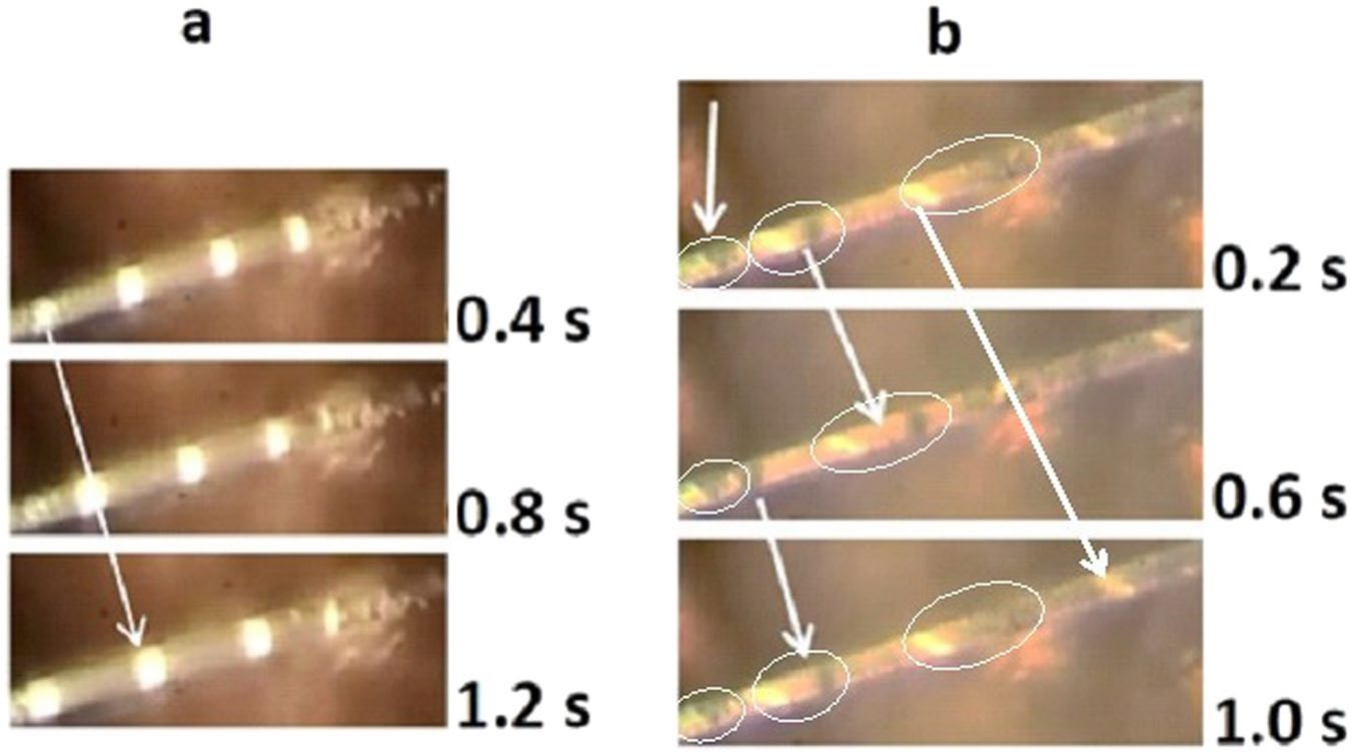
Sliding domains in twinned needle-like VO_2_ single crystal D6(7). (**a**) Three images of single domains (yellow) snipped from a video at 0.4 s intervals (see video clip for I = 0.623 mA) and (**b**) three images of pairs of domains (yellow and black) snipped from a video at 0.4 s intervals (see video clip for I = 0.44 mA). In (**b**) pairs of domains (static at the left of images) and sliding (further along the sample) are surrounded by ellipses.Vertical arrow at top of (**b**) points to a pair of static triangular domains; the arrow in the middle connects the images of this pair at 0.2 s and 0.6 s; the lowest arrow connects the image of a dark domain being emitted by the static domains at 0.6 s with its image at 1.0 s; the long arrow at the right of (b) connects the images of a yellow domain at 0.2 s and 1.2 s.

A rather different scenario in the mixed M-I state was exhibited by a needle-like twinned crystal from the same batch, D6(7). Investigation of this crystal started with I-V characteristics and ended with R(T) measurements. This sample provided the most surprising and intriguing dynamic domain patterns observed so far in VO_2_ single crystals. In ranges of currents the mixed state consisted of emission of sliding single domains while in others it consisted of sliding twin domains emitted by static twin domains. Images of domains, of one type (bright) or of two types (dark and bright) sliding along metallic background are seen in Fig. 4(a,b), respectively. Figure 4(a) represents the usual case in which sliding domains are emitted from a static, unstable I-M boundary^2^. Figure 4(b) shows the most unusual and unexpected case: here, a pair of static, triangular domains, oppositely oriented emit pairs of sliding twins. Three images snipped from the video at three instances, 0.2, 0.6 and 1.0 seconds, follow this event. The vertical arrow at the top of this Figure points to the pair of static triangular twins (yellow and black); to the right of this arrow there is a pair of domains that have just been emitted by them. The arrow in the middle connects the images of this pair at 0.2 s and at 0.6 s: it shows that during this time both domains have moved to the right while the distance between them has increased. The lowest arrow connects the image of a dark domain being emitted by the static domains with its image at 1.0 s; at the latter instance the dark domain is followed, at a distance, by a yellow twin. This Figure shows that while the static triangular twins remain attached, probably releasing strain, pairs of sliding domains repel each other until, with time, a constant distance separates them. In this sample sliding was regular and the domains’ emission frequency, f, and sliding velocity, u, could be measured over a range of currents in the mixed state.

**Figure 5:**
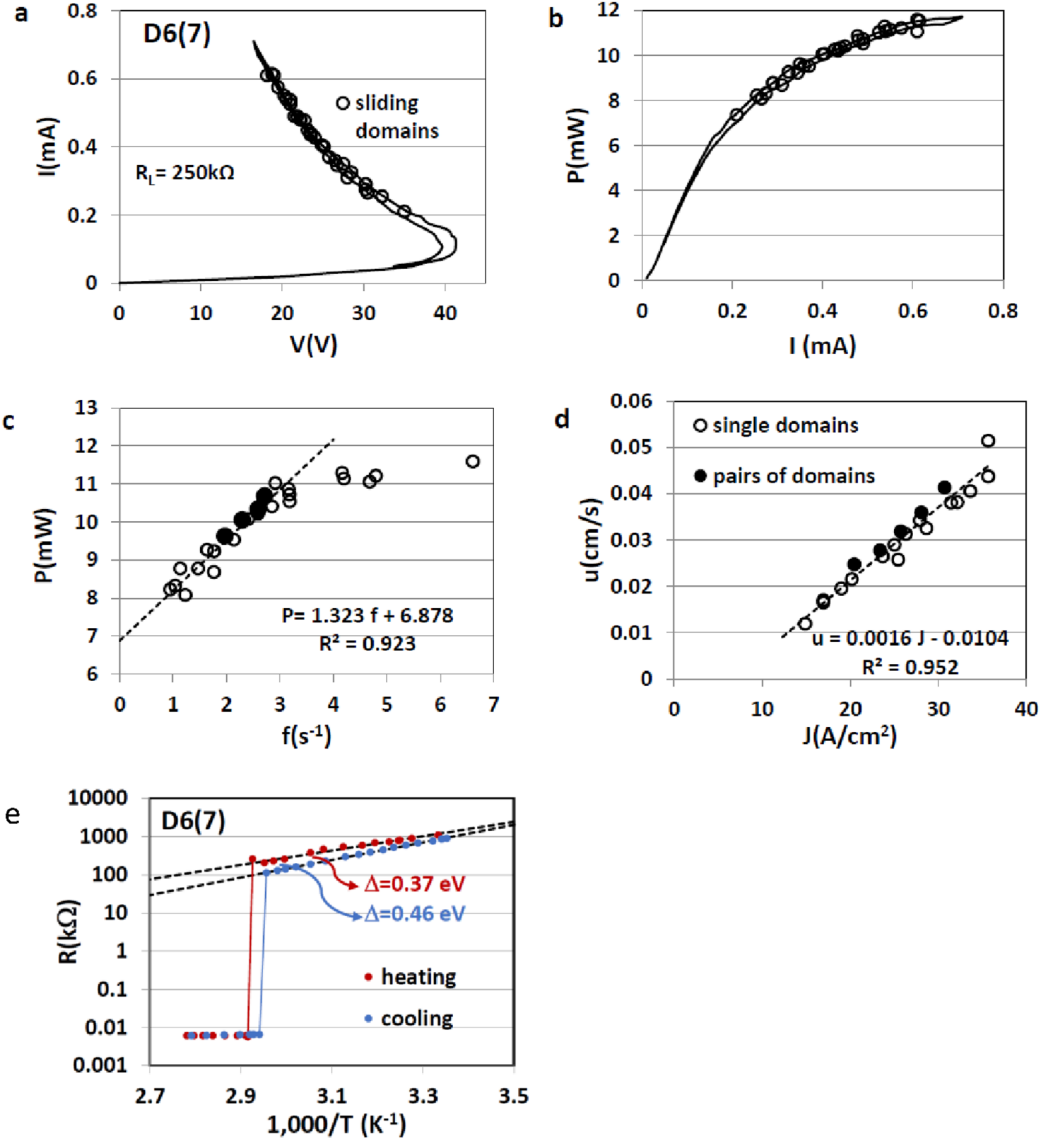
The mixed Metal-Insulator state in twinned needle-like VO_2_ single crystal D6(7). (**a**) I-V characteristic, (**b**) Power (P) versus current (I), (**c**) P versus emission frequency (**f**) and (**d**) sliding velocity (u) versus current density (J) for single and pairs of sliding domains (see Fig. 4), (**e**) Resistance (R) versus inverse temperature.

The I-V characteristic of D6(7) measured under steady state conditions [Fig. 5(a)] shows that the transition to the mixed state is smooth but, although the sample is fairly thin, there is some thermal hysteresis. The bending of P(I) [Fig. 5(b)] is steep at the onset of the mixed state but becomes gradual with increasing currents. There are two types of symbols in Fig. 5(c) for P(f) and 5(d) for u(J), empty and full circles representing single and twin sliding domains, respectively. P(f) in Fig. 5(c) shows that dP/df = ΔE, where ΔE is the energy per domain emission, is constant at low frequencies but above f ≈ 3 s^−1^ it gradually decreases. Within the experimental uncertainty the sliding velocity, u(J), increases at a constant rate du/dJ = Π/L = 0.0016 cm^3^/As which is one of the highest values obtained so far for sliding domains in VO_2_ single crystals.

The semi log plot of R versus 1000/T for sample D6(7) shown in Fig. 5(e) resembles that shown in Fig. 2(a) for sample D6(6b). Upon heating, the activation energy in the insulating regime is low [0.35 eV for D6(6b) and 0.37 eV for D6(7)] and upon cooling it is high [0.45 eV for D6(6b) and 0.46 eV for D6(7)].

The main properties of the twinned samples D6(6a), D6(6b) and D6(7), the main results of measurements and notable observations are summarized in Table 1.

Following the above set of measurements, dark and bright sliding domains were observed in the mixed state of an additional VO_2_ crystal from another batch. In that case a long series of dark domains followed bright ones, or they appeared having various periodicities (one bright followed by one dark or two bright followed by one dark, etc.). However, there the static emitting I-M boundary could not be resolved.

Eight decades after its discovery^14^ the metal-insulator transition in VO_2_ continues to provide, at times, new surprises of fundamental nature that may become useful in applicative research^15^. The present report enriches the known collection of static and dynamic patterns of the mixed M-I phase of VO_2_ single crystals with new intriguing configurations obtained due to twinning.

## Methods

### Materials and Techniques

Single crystals of VO_2_ were grown by self-flux-evaporation^16,17^ from 99.99% V_2_O_5_ powder (Sigma Aldrich). For this study, needle-like crystals with small rectangular cross-section (<200 μm^2^) were chosen from the growth batch. Each batch is labeled Dn1(n2) where n1 is the batch number and n2 the sample number. Samples that cracked or split were discarded at various stages of investigations from very early to fairly advanced unless, as in the case of D6(6), they provided the opportunity to compare different sequences of measurements in almost identical samples.

R(T) and I(V) were measured in the four-probe-two-contacts configuration using indium-amalgam dots for contacts. These are Ohmic contacts of low resistance for insulating (semiconducting) VO_2_. These contacts allow the samples to move freely, they being held only by surface tension. The duration of a full heating-cooling cycle between room temperature and 370 K was about 4 hours.

I-V characteristics were carried out either prior or after R(T) measurements, under steady state conditions (R_L_ ≥ | dV/dI|_max_) in the NDR regime. The I-V loops were recorded on a YEW type 3036X-Y recorder. The duration of a full I-V cycle was about 10 minutes due to the many pauses for video recordings in the mixed state. (Each pause was 20–30 seconds).

The samples were viewed by a Zeiss Stereo Microscope 47 50 57. The videos were recorded using the camera of a smartphone and processed using the movie maker software. The sliding domains emission frequency (f) consisted of counting the number of domains crossing a fixed point in the sample divided by the time interval of the video (only events for which emission persisted during the whole interval were counted); the estimated error of the frequency was 1/20 s^−1^ (and is included in the diameters of the circles in the graphs). The irregularity of emission reduced the R^2^ of the fitted trend-lines of P(f). The domain’s sliding velocity was obtained by measuring their travelling time, Δt, between two fixed points in the sample separated by a distance Δx (u = Δx/Δt); each data point represents the average of u for at least 5 consecutive domains that travelled the same distance at a given current density.

### Polarization by reflection, Brewster angle and extinction

The initial purpose of the investigation reported here was to reach the optimal orientation of the sample under the microscope for maximal contrast between the sliding insulating domains and the metallic background, taking advantage of polarization by reflection (without using polarizers). According to Fresnel’s equations^9^, if light strikes the interface between two isotropic media at the Brewster angle^8^, so that there is a 90 ° angle between the reflected and refracted rays, the reflected light is linearly polarized in a direction parallel to the plane of the interface. Extinction occurs when the incidence angle is the Brewster angle and the incident wave is polarized parallel to the incidence plane^18^.

## Supplementary information

Supplementary information

Supplementary information2 

Supplementary information3 
